# Replication study of significant single nucleotide polymorphisms associated with myopia from two genome-wide association studies

**Published:** 2011-12-16

**Authors:** Qin Wang, Yang Gao, Panfeng Wang, Shiqiang Li, Xiaoyun Jia, Xueshan Xiao, Xiangming Guo, Qingjiong Zhang

**Affiliations:** State Key Laboratory of Ophthalmology, Zhongshan Ophthalmic Center, Sun Yat-sen University, Guangzhou, China

## Abstract

**Purpose:**

Two previous genome-wide association studies (GWAS) of high myopia in a Japanese population found several single nucleotide polymorphisms (SNPs) associated with the disease. The present study examined whether these markers are associated with myopia in a Chinese population.

**Methods:**

Individuals with or without complex myopia were recruited from Chinese university students, and probands with early onset high myopia were identified in the Pediatric and Genetic Eye Clinic of the Zhongshan Ophthalmic Center. DNA was prepared from venous leukocytes. Three SNPs, rs577948 and rs11218544 at chromosome position 11q24.1 and rs2839471 at chromosome position 21q22.3, were genotyped. The allele and genotype frequencies of these SNPs were compared between the myopia cases and controls using a χ^2^ test.

**Results:**

A total of 2,870 subjects were examined in this study, including 1,255 individuals with complex myopia (−10.00 diopter (D)<spherical refraction≤-4.00 D), 563 with early onset high myopia (spherical refraction≤-6.00 D), and 1,052 healthy controls (−0.50 D≤spherical equivalent≤ +2.00 D). There were no statistically significant differences found for the genotype or allele frequencies of the three SNPs between the myopia cases and controls in the Chinese population under study.

**Conclusions:**

We did not find evidence for the association of myopia with rs577948, rs11218544, or rs2839471 in the Chinese population studied.

## Introduction

Myopia is the most common cause of vision impairment worldwide, with a prevalence of approximately 25.4%–80% [1–3]. The prevalence of myopia reaches 71%–96% in students living in the developed region of East Asia [4,5] and is expected to increase [6–8]. Both environmental and genetic factors are believed to play important roles in the development of myopia, but the exact molecular basis of this condition is still unknown [1,9].

Molecular genetic studies have identified several loci associated with a predisposition to myopia [10–27]. However, the genes at most of these loci remain to be identified [28,29]. In the past three years, several independent genome-wide association studies (GWAS) have focused on the search for genetic factors contributing to myopia [20,30–34]. Two of these studies were based on Japanese populations, and identified several single nucleotide polymorphisms (SNPs) significantly associated with high myopia, including rs577948 and rs11218544 at chromosome 11q24.1 and rs2839471 at chromosome 21q22.3 [20,30]. Replication is an important step to confirm the original findings of association studies; although the original reports suggested replication studies to validate their findings, these have yet to be performed.

Both Chinese and Japanese populations exhibit a very high prevalence of myopia. Additionally, the populations of these two regions are closely related [35]. The LD structures around rs577948, rs11218544, and rs2839471 are very similar between Chinese and Japanese populations according to data from HapMap Phase 3. Therefore, we performed a replication study to examine whether the SNPs rs577948, rs11218544, and rs2839471 are also associated with myopia in Chinese individuals.

## Methods

### Subjects

A total of 2,870 unrelated individuals were examined in this study, including 1,052 healthy controls and 1,818 myopia cases. Based on degrees of spherical refraction, the 1,818 subjects with myopia were classified into three groups: −6.00 diopter (D)<spherical refraction≤-4.00 D for 423 cases, −9.25 D<spherical refraction≤-6.00 D for 1,124 cases, and spherical refraction≤-9.25 D (−9.25 D was the criterion for myopia used in the original study [20]) for 271 cases. The 1,818 myopia cases were divided into two groups based on genetic contribution: 563 cases with early onset high myopia and 1,255 cases with complex myopia. The 563 individuals with early onset high myopia were identified at the Pediatric and Genetic Clinic of the Zhongshan Ophthalmic Center. The enrollment criteria used were basically the same as previously described [15]: 1) myopia was present before attending primary school; 2) spherical refraction ≤-6.00 D; 3) no other known ocular or systemic diseases. The 1,255 subjects with complex myopia and 1,052 healthy controls were college students recruited from 12 universities in Guangzhou. The 1,255 subjects with myopia met the following criteria: 1) spherical refraction at each meridian ≤-4.00 D; 2) best corrected visual acuity (BCVA) ≤0.1 in logMAR; 3) myopia onset occurred after 7 years of age; 4) no other known eye or related systemic diseases were present; 5) no family history of high myopia existed. The 1,052 subjects in the healthy control group met the following criteria: 1) bilateral refraction measured between −0.50 D and +2.00 D (spherical equivalent); 2) best unaided visual acuity ≤0 in logMAR; 3) no other known eye or related systemic diseases were present; 4) no family history of high myopia was documented.

The results of ophthalmologic examinations were recorded, including visual acuity (unaided, near, and/or best), color vision, slit lamp and direct ophthalmoscope examination. Refractive errors were measured using an autorefractor (Topcon KR-8000, Paramus, NJ) after mydriasis with compound tropicamide (Mydrin®-P, Santen Pharmaceutical Co. Ltd., Osaka, Japan). All of the college students recruited from 12 universities in Guangzhou received an ocular biometry examination using IOL master V5 (Carl Zeiss Meditec AG, Jena, Germany). Additional examinations included an electroretinogram and fundus photograph in selected individuals. The study adhered to the tenets of the Declaration of Helsinki and was approved by the Institutional Review Board of Zhongshan Ophthalmic Center, Guangzhou China. As this study was part of a project with the aim of identifying genetic factors related to myopia, the procedure for obtaining informed consent and collecting subjects was the same as previously described [36]. Genomic DNA was prepared from venous blood samples obtained from the study subjects.

### Genotyping

Restriction fragment length polymorphism (RFLP) analysis was used to detect the SNP rs2839471. The primers that were used to amplify the DNA fragment that included rs2839471 [provided by the National Center for Biotechnology Information (NCBI)] are listed in Table 1. The amplicon was digested using the restriction endonuclease Hpy188I according to the manufacturer’s instructions (New England Biolabs [Beijing], Ltd, Beijing, China]. The digested products were analyzed by PAGE.

**Table 1:** Primers used for genotyping

**SNP**	**Direction**	**Primer sequence (5′-3′)**
rs2839471	F	TGGGGCTGCAGGTGTTGTG
	R	AGTGGGCCAGCTAGGAAAAGAAA
rs577948	Nest-F	CACAATGCAAAGGATCAGAGC
	Nest-R	AGCGTGATCAGAATACAAGATGG
	F-A	ACTGTCAGAAACTCAACTCTCA
	F-G	TATAACTGTCAGAAACTCAACTCTCG
	R-Pub+M13	CGTTGTAAAACGACGGCCAGTatcagaatacaagatggagcgtagg
rs11218544	Nest-F	GCAAAGGAAACAATCAACAGAG
	Nest-R	AAAGGTGTCCAAATGATAGCC
	F-T	CAAATCACTAACCTTTCCAGAACAT
	F-G	TATACAAATCACTAACCTTTCCAGAACAG
	R-Pub+M13	CGTTGTAAAACGACGGCCAGTggaccaaagacagctcaaacc
M13 probe		FAM-CGTTGTAAAACGACGGCCAGT

The SNPs rs577948 and rs11218544 were genotyped by nested polymerase chain reaction (nest-PCR) combined with fluorescence-labeled allele-specific PCR (AS-PCR). Briefly, two pairs of primers were used. The first pair of primers was designed to amplify fragments encompassing the SNPs. The amplicons were then used as templates for a second round of PCR, in which three internal primers and a labeled common primer were used: two SNP allele-specific primers with a length difference of 4 bp, a reverse specific primer with an M13 tail, and a 5′-FAM-labeled M13 probe (Table 1). Finally, the labeled allele-specific amplicons were analyzed using capillary electrophoresis in an ABI 3100 genetic analyzer (Applied Biosystems, Foster City, CA) and analyzed using GeneMapper software v3.5 (Applied Biosystems).

For each SNP, 48 samples were simultaneously analyzed by cycle sequencing to ensure that either the RFLP or M13-tailed method was reliable. Genotyping results were read independently by two researchers. Two types of additional validation were conducted: 1) direct sequencing of the first round of PCR products from four samples in each plate (96 samples); and 2) any sample that was read differently by the two researchers was confirmed by sequencing.

### Statistical analysis

The distribution of sexes was compared between the myopia cases and healthy controls with a χ^2^ test. The average age was compared between the myopia cases and healthy controls with a *t*-test. The distribution of all three SNPs was evaluated with respect to Hardy–Weinberg equilibrium (HWE). The frequencies of alleles and genotypes in the early onset high myopia and complex myopia groups, as well as in the classified spherical refraction groups were compared with those in the healthy control group using a χ^2^ test. A p<0.05 was used as the level of statistical significance (α), and a Bonferroni correction was applied for multiple testing, correcting the threshold for significance from 0.05 to 0.017 (α/3=0.05/3).

## Results

**Figure 1:**
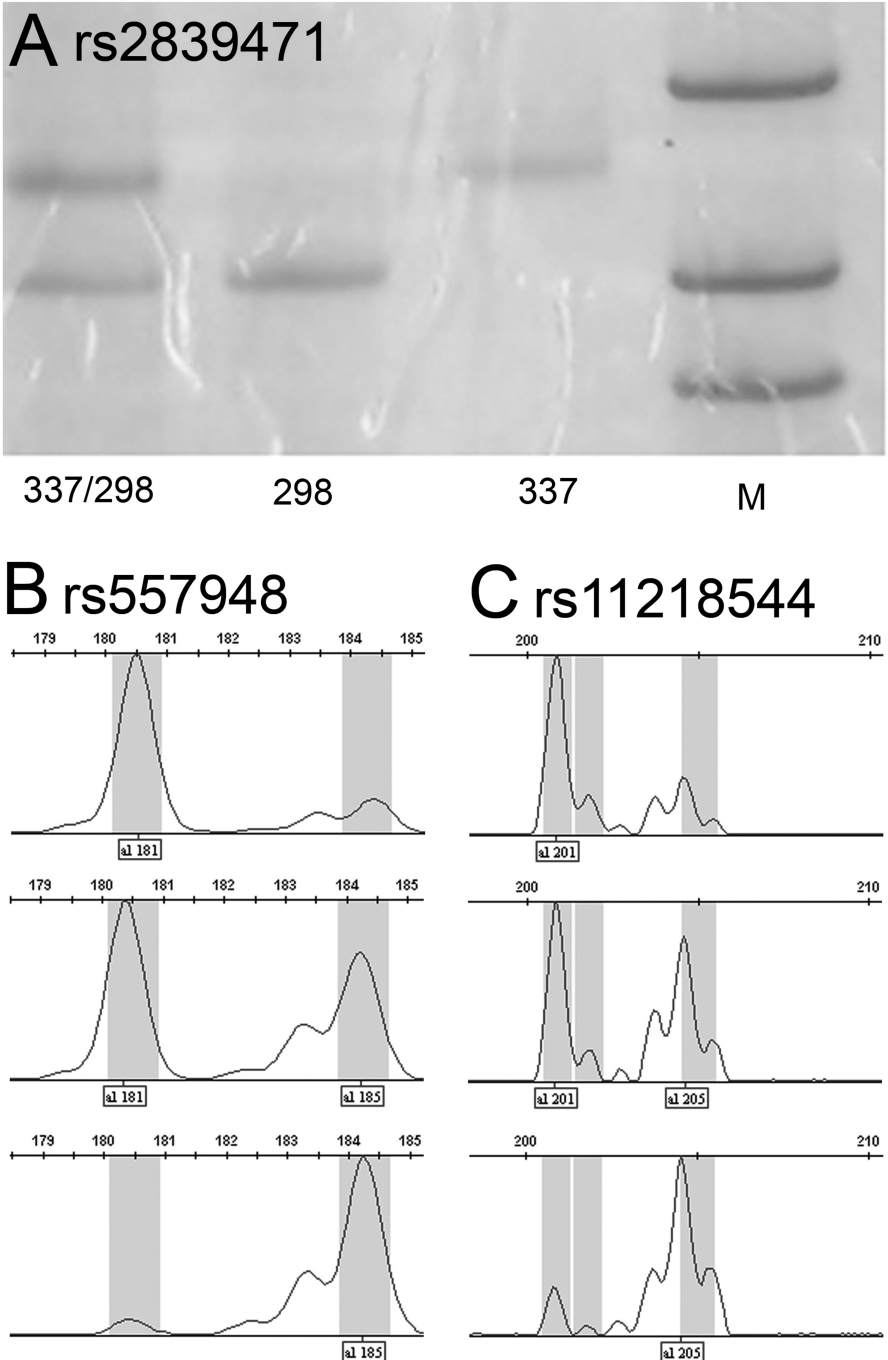
Genotyping of rs2839471, rs577948, and rs11218544. The three SNPs were successfully genotyped in 2,870 subjects. M: 100 bp DNA ladder. **A**: RFLP analysis of SNP rs2839471. The double bands at 337 bp and 298 bp represent genotype C/T, the single band at 298 bp represents C/C, and the single band at 337 bp represents T/T. Capillary electrophoresis analysis was used for genotyping rs577948 and rs11218544. The three peak patterns represent three different SNP genotypes. **B**: SNP rs577948: the single peak at 181 bp is genotype A/A, the double peaks at 181 bp and 185 bp are A/G, and the single peak at 185 bp is G/G. **C**: SNP rs11218544: the single peak at 201 bp is T/T, the double peaks at 201 bp and 205 bp are T/G, and the single peak at 205 bp is G/G.

The basic clinical information for all 2870 subjects is listed in Table 2. Genotyping of rs2839471, rs577948, and rs11218544 was successfully performed in the 2,870 unrelated subjects (Figure 1, Table 3, and Table 4). The genotyping success rate was 100% for the three SNPs. There was no significant deviation from HWE for any of the three SNPs in the healthy controls. No statistically significant difference in genotype frequencies or allele frequencies was found between the myopia cases and controls, regardless of whether myopia was classified into early onset high myopia and complex myopia or classified into three groups based on spherical refraction (Table 3 and Table 4). Although the frequency of rs11218544 exhibited a slight difference between the complex myopia group and healthy control group (allele p=0.032), the difference was not significant after Bonferroni correction with a corrected significance level of p=0.017.

**Table 2:** Basic information for the 2,870 subjects.

**Category**		**Early onset high myopia**		**Complex myopia**			**Healthy control**
		**−9.25 D<S≤-6.00 D**	**S≤-9.25 D**	**−6.00 D<S≤-4.00 D**	**−9.25 D<S≤-6.00 D**	**S≤-9.25 D**	**−0.50 D≤SE≤+2.00 D**
Number of subjects		355	208	615	610	30	1052
Age (years)	Range	0.3~79	2~64	16~27	15~33	19~27	16~32
	Average	13.44±9.28	20.06±14.01	20.70±1.59	20.85±1.73	20.94±1.53	20.91±1.78
Gender	Male	0.535	0.474	0.503	0.476	0.486	0.597
	Female	0.465	0.526	0.497	0.524	0.514	0.403
Refraction (D)	Range	−6.00~-9.00	−30.00~-9.25	−4.00D~-5.87D	−6.00D~-9.00D	−10.00~-9.25	−0.5~+2.00
	Average	−7.70±0.95	−14.46±4.51	−5.06±0.52	−7.00±0.88	−9.64±0.31	0.25±0.52
Axial length (mm)	Range	not available	not available	23.39~28.36	24.00~29.30	25.29~29.32	20.30~26.53
	Average			25.79±0.81	26.48±0.91	27.29±0.83	23.60±0.79

Abbreviations: S,spherical refraction; SE,spherical equivalent.

**Table 3:** Allele frequency and genotypes of the three SNPs in myopia cases and healthy controls. Myopia was classified as early onset high myopia and complex myopia.

**SNPs**	**Allele (A/B)**	**Classification of subjects**		**Number of subjects**	**MAF**	**Genotype**			p value*		**OR**	**95%CI**
						**A/A**	**A/B**	**B/B**	**Allele**	**Genotype**		
rs2839471	C/T	myopia	complex (−10.00D≤S≤-4.00D)	1255	0.489	293	641	321	0.393	0.266	1.052	0.937–1.181
			early onset (S≤-6.00D)	563	0.472	116	300	147	0.838	0.131	0.985	0.852–1.139
			complex+early onset	1818	0.484	409	941	468	0.582	0.151	1.031	0.926–1.148
		healthy control	−0.50D≤SE≤+2.00D	1052	0.476	248	506	298				
rs577948	A/G	myopia	complex (−10.00D≤S≤-4.00D)	1255	0.494	302	665	288	0.307	0.269	1.062	0.946–1.193
			early onset (S≤-6.00D)	563	0.491	123	307	133	0.973	0.282	1.003	0.867–1.159
			complex+early onset	1818	0.499	425	972	421	0.439	0.207	1.043	0.937–1.162
		healthy control	−0.50D≤SE≤+2.00D	1052	0.49	251	530	271				
rs11218544	G/T	myopia	complex (−10.00D≤S≤-4.00D)	1255	0.401	200	607	448	0.032	0.098	1.14	1.012–1.284
			early onset (S≤-6.00D)	563	0.382	83	264	216	0.515	0.771	1.051	0.905–1.220
			complex+early onset	1818	0.395	283	871	664	0.061	0.17	1.112	0.995–1.242
		healthy control	−0.50D≤SE≤+2.00D	1052	0.37	142	495	415				

Note: MAF=minor allele frequency; S=spherical refraction; SE=spherical equivalent. *P value was calculated by chi-square test between myopia and normal control.

**Table 4:** Allele frequency and genotypes of the three SNPs in myopia cases and healthy controls. Myopia was classified based on the degrees of refractive errors.

**SNPs**	**Allele (A/B)**	**Classification of subjects**		**Number of subjects**	**MAF**	**Genotype**			p value*		**OR**	**95%CI**
						A/A	A/B	B/B	Allele	Genotype		
rs2839471	C/T	myopia	−6.00D<S≤-4.00D	615	0.497	153	313	149	0.132	0.19	1.114	0.968~1.283
			−9.25D<S≤-6.00D	965	0.478	212	499	254	0.899	0.269	1.008	0.891~1.141
			S≤-9.25D	238	0.456	44	129	65	0.422	0.153	0.921	0.755~1.125
		Healthy control	−0.50D≤SE≤+2.00D	1052	0.476	248	506	298				
rs577948	A/G	myopia	−6.00D<S≤-4.00D	615	0.497	152	315	148	0.477	0.735	1.052	0.914–1.211
			−9.25D<S≤-6.00D	965	0.499	216	531	218	0.591	0.099	1.034	0.914–1.171
			S≤-9.25D	238	0.496	57	126	55	0.589	0.673	1.056	0.866–1.289
		Healthy control	−0.50D≤SE≤+2.00D	1052	0.49	251	530	271				
rs11218544	G/T	myopia	−6.00D<S≤-4.00D	615	0.402	103	289	223	0.065	0.148	1.146	0.992–1.323
			−9.25D<S≤-6.00D	965	0.397	153	460	352	0.082	0.21	1.119	0.986–1.271
			S≤-9.25D	238	0.37	27	122	89	0.984	0.446	0.998	0.812–1.226
		Healthy control	−0.50D≤SE≤+2.00D	1052	0.37	142	495	415				

Note: MAF=minor allele frequency; S=spherical refraction; SE=spherical equivalent. *P value was calculated by χ^2^ test between myopia and normal control.

## Discussion

In this study, we genotyped three SNPs that were previously reported to be significantly associated with myopia. The SNP rs2839471 is located in the region of the uromodulin-like 1 (*UMODL1*) gene, which may be associated with extracellular matrix and involved in the formation of sclera [37,38]. The SNPs rs577948 and rs11218544 are located in a region that includes two genes, BH3-like motif containing protein (*BLID*) and *LOC39959*. *BLID* is expressed in mitochondria, and may function in apoptosis in pathological myopia [39–41]. *LOC399959* is a hypothetical noncoding RNA [30]. Further study may elaborate the function of these genes. In the previous Japanese studies, axial length or spherical equivalent were used as criteria for recruiting the myopia patients. The proportion of early onset myopia or complex myopia was not reported [20,30]. In this study, no significant association was found between myopia and the three SNPs examined, regardless of whether myopia was classified based on genetic contribution (early onset high myopia and complex myopia) or based on degrees of spherical refraction. Therefore, our results suggest that these three SNPs may not be associated with myopia in the Chinese subjects we studied. Currently, multiple association studies are inconsistent and the causes of these contradictions have been widely analyzed [42,43]. The reasons why our results in the Chinese population were not identical to the Japanese results are likely related to the recruitment criteria, environmental influences, and genetic differences between the Chinese population and Japanese population because an earlier Chinese replication of a Japanese study on chromosome 11q24.1 (rs577948) also failed to confirm the reported association [44]. Furthermore, another explanation may be that the genetic variants interact with one another, and the interaction between genes and environmental factors vary in different human populations, although this has not been systematically assessed [45]. Although our results do not support the previous findings related to these SNPs [20,30], the results obtained with a Japanese population cannot be considered false-positives at this stage. However, this result must be analyzed with great caution until additional replication studies confirm a reported association, especially for myopia.

The distribution of sexes was not completely matched in the myopia and healthy control groups. However, this does not affect our conclusions because the results were unaffected when male subjects were randomly excluded to reach a balanced proportion of sexes. In addition, on average our study subjects were younger than those in the Japanese study for both cases and controls. This should not influence our conclusion because myopia is an early onset disease, and the degree of refractive error in myopia usually increases with age [46–48]. In our study females are more myopic (odds ratio>1, p<0.05) than males, which is consistent with previous reports [49,50]. On the other hand, males with a higher prevalence of myopia or no difference among genders were also reported [45,51,52]. Lower age as a predictor of myopia (odds ratio<1,p<0.001) was also consistent with results reported in other populations [53,54].

Myopia is a genetically heterogeneous disease, and both early onset and complex traits have been suggested to be associated with myopia. Traditionally, myopia has generally been classified based on degrees of spherical refraction or their closely related axial length [20,30]. In this study, we attempted to classify myopia based on either the type of genetic contribution, or based on degrees of spherical refraction. We did not find a significant association between the SNPs analyzed and myopia in either case. Additional studies are expected to validate the original findings [20,30].
